# Tracing the Evolution of Sex Hormones and Receptor‐Mediated Immune Microenvironmental Differences in Prostate and Bladder Cancers: From Embryonic Development to Disease

**DOI:** 10.1002/advs.202407715

**Published:** 2025-02-25

**Authors:** Dengxiong Li, Zhipeng Wang, Qingxin Yu, Jie Wang, Ruicheng Wu, Zhouting Tuo, Koo Han Yoo, Dilinaer Wusiman, Luxia Ye, Yiqing Guo, Yubo Yang, Fanglin Shao, Ziyu Shu, Uzoamaka Okoli, William C. Cho, Wuran Wei, Dechao Feng

**Affiliations:** ^1^ Department of Urology Institute of Urology West China Hospital Sichuan University Chengdu 610041 China; ^2^ Department of Urology Sichuan Provincial People's Hospital University of Electronic Science and Technology of China Chengdu 610041 China; ^3^ Department of pathology Ningbo Clinical Pathology Diagnosis Center Ningbo City Zhejiang Province 315211 China; ^4^ Department of Urological Surgery Daping Hospital Army Medical Center of PLA Army Medical University Chongqing 404100 China; ^5^ Department of Urology Kyung Hee University Seoul 04510 South Korea; ^6^ Department of Comparative Pathobiology College of Veterinary Medicine Purdue University West Lafayette IN 47907 USA; ^7^ Purdue Institute for Cancer Research Purdue University West Lafayette IN 47907 USA; ^8^ Department of Public Research Platform Taizhou Hospital of Zhejiang Province Affiliated to Wenzhou Medical University Linhai 317000 China; ^9^ Department of Urology Three Gorges Hospital Chongqing University Wanzhou Chongqing 404000 China; ^10^ Department of Rehabilitation The Affiliated Hospital of Southwest Medical University Luzhou 646000 P. R. China; ^11^ Department of Earth Science and Engineering Imperial College London London SW7 2AZ UK; ^12^ Joint International Research Laboratory of Green Buildings and Built Environments (Ministry of Education) Chongqing University Chongqing 400045 China; ^13^ Division of Surgery & Interventional Science University College London London W1W 7TS UK; ^14^ Basic and Translational Cancer Research Group Department of Pharmacology and Therapeutics College of Medicine University of Nigeria Enugu State Nsukka 410001 Eastern part of Nigeria; ^15^ Department of Clinical Oncology Queen Elizabeth Hospital Hong Kong SAR 999077 China

**Keywords:** bladder cancer, developmental biology, immune microenvironment, prostate cancer, receptors, sex hormones

## Abstract

The bladder and prostate originate from the urogenital sinus. However, bladder cancer (BC) is usually classified as an immune “hot” tumor, whereas prostate cancer (PCa) is deemed as an immune “cold” tumor according to the tumor microenvironment (TME) and clinical outcomes. To investigate the immune differences between BC and PCa, studies are compared focusing on immune regulation mediated by sex hormones and receptors to identify key genes and pathways responsible for the immune differences. From a developmental perspective, it is shown that PCa and BC activate genes and pathways similar to those in the developmental stage. During prostate development, the differential expression and function of the androgen receptor (AR) across cell types may contribute to its dual role in promoting and inhibiting immunity in different cells. Androgen deprivation therapy affects AR function in different cells within the TME, influencing immune cell infiltration and antitumor function. Additionally, estrogenα and estrogenβ exert contrasting effects in PCa and BC, which may hold the potential for modifying the “cold” and “hot” tumor phenotypes. Future research should target key genes and pathways involved in bladder development to clarify the immune regulatory similarities and differences between BC and PCa.

## Graphical abstract

Prostate and bladder cancers activate genes and pathways similar to those in the developmental stage by aggregating and analyzing current data. In this process, sex hormones and their receptors maybe a key role in causing and reversing the “cold” and “hot” immune tumor phenotypes, potentially enhancing the immune response.

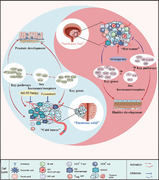

## 1 Introduction

The bladder and prostate originate from the urogenital sinus (UGS), which has both endodermal and mesodermal origins during the embryonic stage.^[^ ^1^ ^]^ Anatomically, the bladder and the prostate are adjacent organs that play crucial roles in urination. However, recent studies have revealed significant differences in the immune status of bladder cancer (BC) and prostate cancer (PCa) in terms of tumorigenesis, cancer progression, and treatments.^[^ ^2^ ^]^ Researchers have gained valuable insights into these differences by exploring various approaches, including those involving sex hormones and their receptors.^[^ ^3^ ^]^ For example, androgen receptor (AR) expression was found to be negatively associated with Programmed Cell Death 1 Ligand 1 (PD‐L1) expression in BC cells, thereby enhancing the antitumor efficacy of immunotherapy.^[^ ^4^ ^]^ In the context of PCa, AR in CD8+ T cells can influence Interferon Gamma (IFN‐γ) protien expression by regulating the transcription of IFN‐γ and Granzyme B (GZMB), which in turn inhibits the function of CD8+ T cells.^[^ ^5^ ^]^ In clinical treatments, significant differences exist, particularly in the field of immunotherapy.^[^ ^6^ ^]^ Many immunotherapies, including anti‐PD1 and anti‐PDL1 drugs, have shown acceptable response rates in BC and can significantly improve patient prognosis.^[^ ^7^ ^]^ Based on these positive outcomes, immunotherapy has been recommended as a treatment option for patients with chemoresistant BC. Current trials assessing the potential of immunotherapy as neoadjuvant therapy, alone or in combination with chemotherapy, to manage BC, have shown promising results.^[^ ^8^ ^]^ The FDA has approved several immune checkpoint inhibitors for the treatment of BC, including atezolizumab, pembrolizumab, avelumab, nivolumab, and durvalumab.^[^ ^9^ ^]^ In contrast to those with BC, patients with PCa exhibit a low response rate to immunotherapy, leading to minimal survival benefits.^[^ ^10^ ^]^ Consequently, the America Food and Drug Administration has only approved pembrolizumab and dostarlimab in a tumor‐agnostic manner for a small subset of patients with metastatic castration‐resistant PCa.^[^ ^11^ ^]^ This disparity may be attributed to the tumor microenvironment (TME) of PCa, which is characterized by a noninflamed state and increased infiltration of immunosuppressive cell subsets.^[^ ^9b^ ^]^ For example, in mouse models of bone metastasis, Transforming Growth Factor Beta 1 released by castration‐resistant prostate cancer (CRPC) cells promotes the development of T helper 17 cells over T helper 1 cells, thereby diminishing immunotherapy efficacy.^[^ ^12^ ^]^ Consequently, BC is often referred to as an immune “hot” tumor, while PCa is classified as an immune “cold” tumor, based on the TME and clinical outcomes. Several studies have explored various approaches to address this issue, focusing on the regulation of sex hormones and their receptors.^[^ ^13^ ^]^ Furthermore, patients with nonmuscle‐invasive bladder cancer (NMIBC) can benefit from intravesical Bacillus Calmette‐Guerin (BCG) therapy; however, ≈30% of these patients do not respond to BCG.^[^ ^14^ ^]^ Mizushima et al.^[^ ^15^ ^]^ reported that AR signaling diminishes the efficacy of BCG treatment by enhancing RAB27B‐induced exocytosis. In the context of PCa, a combination of androgen deprivation therapy (ADT) and atezolizumab has been shown to improve radiographic progression‐free survival in patients with metastatic castration‐resistant PCa.^[^ ^16^ ^]^ These findings suggest that sex hormones and their receptors play significant roles in drug resistance by modulating the immune microenvironment.

An intriguing question arises: why do these two organs, despite similar developmental processes, close anatomical proximity, and overlapping functions, exhibit distinct differences in immune response? Recent research has shown that the initiating cells of pediatric brain tumors closely resemble specific developmental brain cell subgroups at the transcriptomic level. This conclusion was drawn from a comparative analysis of transcriptomic profiles during organ development and tumor initiation.^[^ ^17^ ^]^ In another study, researchers performed single‐cell sequencing comparisons of the human fetal liver, hepatocellular carcinoma, and mouse liver, revealing that the tumor microenvironment displays fetal‐like reprogramming. This phenomenon included the re‐emergence of fetal‐associated endothelial cells (PLVAP/VEGFR2) and fetal‐like tumor‐associated macrophages (FOLR2).^[^ ^18^ ^]^ Consequently, the dedifferentiation observed during tumor development and progression appears to exhibit characteristics reminiscent of a reverse organ development process.^[^ ^19^ ^]^ These compelling studies highlight the relationship between the developmental microenvironment and TME and inspired us to investigate the immune differences between BC and PCa by comparing the developmental microenvironments and TME of the bladder and prostate.

Understanding the differences between BC and PCa in terms of their developmental microenvironments, carcinogenesis, and progression can offer insights into the mechanisms underlying the observed immune disparities, particularly from the perspective of key genes and signaling pathways. During the embryonic and pubertal stages, prostate growth and differentiation require the involvement of sex hormones, whereas bladder development does not.^[^ ^20^ ^]^ Unlike the mature prostate, which secretes fluids integral to reproduction, the bladder does not have a reproductive function. These differences in sex hormones and receptor levels may represent the most significant differences between BC and PCa. Therefore, we analyzed and compared studies that focused on immune regulation mediated by sex hormones and receptors to identify the key genes and pathways responsible for the immune differences between BC and PCa. This understanding could provide insights into the mechanisms underlying these differences and highlight potential approaches for modulating the immune environment. These insights may be crucial for developing improved immunotherapy strategies for both BC and PCa.

## 2 The Development Process of Prostate and Bladder

The UGS begins to develop during gestational week (GW) 7 in humans.^[^ ^21^ ^]^ The UGS consists of the urogenital sinus epithelium. Subsequently, androgens secreted by the Leydig cells of the embryonic testis initiate prostate specification by activating AR expression.^[^ ^22^ ^]^ Prostate epithelial progenitor cells express KRT5, KRT8, and TRP63. During this process, prostate‐specific antigen (PSA) is secreted by luminal cells during GW15.^[^ ^23^ ^]^ In mature prostate tissue, epithelial cells comprise luminal cells (expressing KRT8, KRT18, and AR), basal cells (expressing KRT5, KRT14, P63, CK8, CK18, and TRP63), and neuroendocrine cells (expressing synaptophysin and chromogranin A [CGA]).^[^ ^24^ ^]^ Furthermore, basal cells can be converted into neuroendocrine cells.^[^ ^25^ ^]^ In this context, ZEB1 serves as a marker of multipotent basal stem cells.^[^ ^26^ ^]^ Interactions between AR and the SHH, BMP, WNT/β‐catenin, SOX9, and TGF‐β pathways significantly influence prostate development. Prostate development in humans and mice is similar in terms of epithelial budding, ductal elongation, and marker expression, except for morphological organization.^[^ ^1^, ^27^ ^]^ The adult human prostate comprises the peripheral, central, and transitional zones, containing 30–50 tubuloalveolar glands.^[^ ^28^ ^]^ The prostate secretes prostatic fluid, a crucial component of semen. In mice, the prostate consists of ventral, dorsal, lateral, and anterior prostate lobes.^[^ ^29^ ^]^

In mice, bladder formation is initiated during late gestation, whereas in humans, it occurs during early gestation.^[^ ^30^ ^]^ At embryonic day (E)11, bladder epithelial progenitor cells initiate growth and differentiation.^[^ ^31^ ^]^ The progenitor cells generate intermediate, basal, and superficial cells.^[^ ^32^ ^]^ K14 basal cells can be divided into intermediate and K5 basal cells. The intermediate cells can produce superficial cells.^[^ ^31^ ^]^ The SHH, BPM4, NOTCH, and WNT/β‐catenin pathways play crucial roles in bladder development. In mature bladder tissue, epithelial cells are categorized into several types: superficial cells (expressing KRT20, FABP4, UPK, PPARG, and ZO1), intermediate cells (expressing P63, SHH, UPK, and PPARG), K5 basal cells (expressing P63, SHH, PPARG, and KRT5), and K14 basal cells (expressing P63, SHH, PPARG, KRT5, and KRT14).^[^ ^31^ ^]^ The mature bladder consists of superficial urothelial cells, a submucosal layer (which includes interstitial cells, fibroblasts, and myofibroblasts), and multiple layers of detrusor muscle and is enveloped by a serous membrane composed of the mesothelium and elastic fibrous connective tissue. The urothelium is multilayered and responsive to stretch.^[^ ^33^ ^]^ AR is expressed in fetal and normal maturation bladders in humans and other animals.^[^ ^34^ ^]^

**Figure 1:**
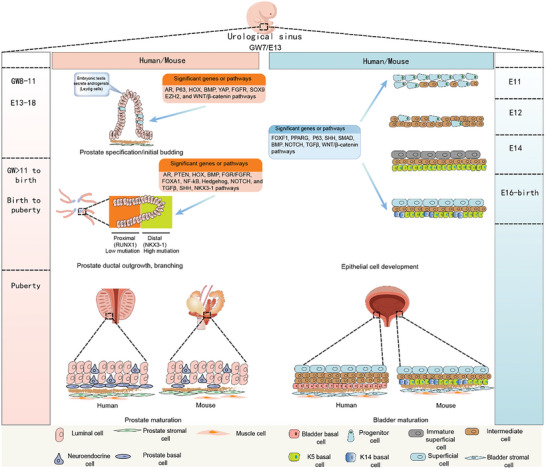
Prostate and bladder developmental process: genes and pathways which play a crucial role in the development of the prostate and bladder; there are some differences between the human and mouse prostate and bladder. E: Embryonic day in mouse; GW: gestational week in human.

Sex hormones and their receptors play crucial roles in the bladder and prostate development. In the context of prostate development, AR expression is essential in stromal cells, including fibroblasts and smooth muscle cells, during prostate bud formation, rather than in epithelial or progenitor cells.^[^ ^26^, ^35^ ^]^ Notably, peri‐epithelial fibroblasts in mice at E17.5, exhibit overexpression of AR, BMP7, WIF1, and WNT5A.^[^ ^26^ ^]^ Similarly, during duct formation, branching, and puberty, AR is required in fibroblasts and smooth muscle cells.^[^ ^36^ ^]^ Interestingly, AR expression activates the prostatic epithelium, differentiating it from the bladder or female urogenital sinus.^[^ ^37^ ^]^ However, AR depletion induced testicular feminization in male mice and blocked prostate formation.^[^ ^38^ ^]^ Furthermore, estrogen and estrogen receptors (ER) are necessary for prostate development; specifically, Erα knockout in epithelial cells or fibroblasts impedes branching morphogenesis.^[^ ^39^ ^]^ Additionally, a high dose of the estrogenic drug diethylstilbestrol completely suppresses the formation of the dorsal and lateral prostate in male fetuses, whereas a low dose of estrogen promotes prostate duct development.^[^ ^40^ ^]^ During bladder development, the AR is expressed in some stromal nuclei in rat embryonic bladders at E14.5.^[^ ^41^ ^]^ Epithelial differentiation of the bladder requires androgen involvement, because AR expression may influence both uroplakin and FOXA2 expression.^[^ ^41^ ^]^ Testosterone also improves bladder weight and structure in male rats.^[^ ^42^ ^]^ In vitro,17b‐estradiol promoted the proliferation of human urothelium (HUC cell line).^[^ ^43^ ^]^ Low doses of estrogen have been associated with malformations in the urethral segment connected to the bladder neck, resulting in urinary retention.^[^ ^40^ ^]^ These results indicate a significant role for sex hormones and receptors, particularly androgens and AR, in the development of the prostate and bladder. In adult organs, AR expression is significantly correlated with carcinogenesis and progression.^[^ ^44^ ^]^ There are several ERs with various functions in bladder and prostate tumorigenesis and progression.^[^ ^40^, ^45^ ^]^ However, there is limited research demonstrating the role of estrogen in bladder and prostate development, which warrants further exploration in future studies. **Figure** **1** presents a concise overview of the developmental processes of the prostate and bladder.

**Figure 2:**
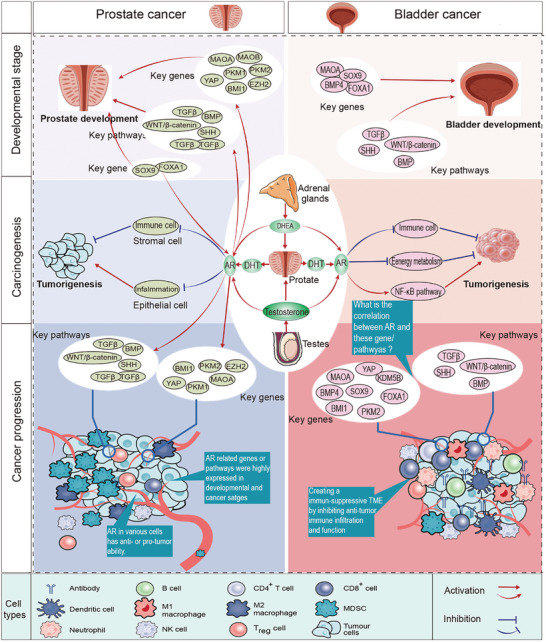
The role of androgens and androgen receptors (AR) in the development, carcinogenesis, and progression of prostate and bladder cancer: similar active genes and pathways between development and cancer progression; the role of AR in bladder development and tumor progression requires further validation; AR exhibits different expression statuses and immunomodulatory functions in various cells of the prostate development duration and prostate cancer stage. AR: androgen receptor; DHEA: dehydroepiandrosterone; DHT: dihydrotestosterone.

## 3 Comparing Androgen and AR Between PCa and BC: An Immunological Perspective

AR/androgen are involved in the development, progression, and treatment of BC and PCa by regulating the immune microenvironment. AR and ADT affected the immune factors and cells to control PCa prognosis.^[^ ^44^, ^46^ ^]^ In BC, the reduced expression of TCF7 and TCF1 in CD8+ T cells mediated by AR may lead to T cell exhaustion, creating an immunosuppressive TME that diminishes the efficacy of immunotherapy.^[^ ^44b^ ^]^ This section delineates and compares the roles of the AR and its regulatory network in PCa and BC, particularly from the perspective of embryonic development.

### 3.1 The Tumorigenesis of the Bladder and Prostate

AR is involved in the whole process of prostate development,^[^ ^26^, ^35^ ^]^ maturation,^[^ ^36^ ^]^ hyperplasia,^[^ ^47^ ^]^ carcinogenesis, and cancer progression, whereas it mainly affects BC development.^[^ ^48^ ^]^ Aging and inflammation contribute to benign prostatic hyperplasia (BPH) and facilitate prostate tumorigenesis.^[^ ^49^ ^]^ In androgen‐induced BPH, AR can modulate oxidative stress levels to regulate BPH by targeting the TGF‐β/NOX4 signaling pathway.^[^ ^50^ ^]^ Additionally, chronic bacterial inflammation reduces AR expression in the prostate tissue and stimulates epithelial cell proliferation, ultimately leading to prostate tumorigenesis.^[^ ^51^ ^]^ The inhibition of AR expression by feedback may result from bacterial activity‐induced inflammation. In BPH tissue, club and hillock cells, located in the proximal region of the prostate ducts and analogous to luminal cells, produce higher levels of immune factors, including antibacterial proteins and chemokines, by upregulating immunomodulatory genes, particularly those associated with the NOTCH pathway, while exhibiting lower AR activation than normal prostate tissue.^[^ ^52^ ^]^ Consequently, prostate inflammation enhances the proliferation of club and hillock cells through IL‐1 secreted by macrophages.^[^ ^53^ ^]^ Furthermore, suppression of AR expression in luminal cells leads to chronic inflammation in prostate tissues by activating macrophage‐generated IL‐1, which is associated with ADT resistance.^[^ ^54^ ^]^ AR expression plays a significant role in stromal cell carcinogenesis. Deletion of AR in fibromuscular cells affects prostate carcinogenesis by reducing the infiltration of immune cells, including T cells and macrophages, thereby inducing a pro‐tumor immune microenvironment in AR knockout mice.^[^ ^55^ ^]^ Similarly, AR knockout in fibroblasts can influence immune cell infiltration through the regulation of MIP‐1α, MIP‐1β, MIP‐2, IL‐10, and IL‐1β expression, and NF‐κB pathway activation, thereby modulating PCa development.^[^ ^55^ ^]^ Furthermore, AR ablation in T cells enhances their antitumor function and systemic T cell response by upregulating antigen recognition, which helps prevent prostate tumorigenesis.^[^ ^56^ ^]^ These findings indicate that AR play different roles in various cell types. In epithelial cells, AR depletion induces inflammation, leading to prostate carcinogenesis. In contrast, in stromal cells, AR expression is positively correlated with tumorigenesis through the regulation of immune cells and their associated factors. As mentioned above, epithelial cells transiently express AR during the embryonic period, whereas AR is essential in stromal cells, primarily fibroblasts and smooth muscle cells. This distinction may fundamentally determine the anti‐ or pro‐tumor functions of AR in different cell types, ultimately influencing the immune microenvironment and treatment efficacy.

During bladder development, AR is expressed in certain stromal nuclei of the E14.5 rat embryonic bladder.^[^ ^41^ ^]^ However, the specific roles of AR in bladder development remain unclear. Notably, serum testosterone levels positively correlate with the incidence of BC.^[^ ^57^ ^]^ Additionally, several studies have indicated that the AR may promote bladder tumorigenesis.^[^ ^58^ ^]^ NF‐kB could enhance the transcription of AR in PCa.^[^ ^59^ ^]^ Similarly, interactions between AR and the NF‐kB pathway have been implicated in promoting bladder tumorigenesis.^[^ ^60^ ^]^ Patients treated with antiandrogen drugs, such as 5‐alpha reductase inhibitors (finasteride), have been associated with a lower incidence of secondary BC.^[^ ^61^ ^]^ The effect of AR expression on CD8+ T cells may explain this phenomenon.^[^ ^62^ ^]^ Mechanistically, AR reduces CD8+ T cell infiltration and diminishes both the stemness and anti‐tumor functions of CD8+ T cells by modulating key pathways and immune factors, including TNF‐α and IFN‐γ. The evidence demonstrates that AR plays an oncogenic role in bladder tumorigenesis by influencing the immune microenvironment while exhibiting distinct functions in various prostate cells. AR primarily promotes carcinogenesis in bladder and prostate tissues by modulating inflammation and fostering a pro‐tumor immune microenvironment. The interaction between the NF‐kB pathway and AR is crucial for bladder tumorigenesis and BC progression. However, the role of the NF‐kB pathway in bladder and prostate development remains to be explored, motivating further investigations in this area. Recently, membrane sex hormone receptors have been shown to influence the occurrence and progression of urological diseases.^[^ ^63^ ^]^ Mechanistically, the interaction between dehydroepiandrosterone and ADGRG2, an adhesion G protein‐coupled receptor, is linked to male fertility.^[^ ^64^ ^]^ Similarly, GPR126, another adhesion G protein‐coupled receptor, is activated by progesterone to promote breast cancer progression via the GI pathway, both in vitro and in vivo.^[^ ^65^ ^]^ These findings suggest the need to further investigate the role of AR in the immune modulation of BC. Additionally, research on the function of AR in bladder development could enhance our understanding of its interactions with the immune system in BC, highlighting the need for further exploration in this field.

### 3.2 The Progression of PCa and BC in Relation to AR‐Related Genes

Numerous AR‐related genes play significant roles in bladder and prostate development. Additionally, these genes influenced immune regulation in BC and PCa. The interaction between the AR and BMI1 notably affects PCa progression and resistance to ADT.^[^ ^66^ ^]^ BMI1+ luminal progenitor cells facilitate the regeneration and self‐renewal of the prostate in mice by activating the IKKα/E2F1/BMI1 axis, which involves the infiltration of B cells.^[^ ^67^ ^]^ In PTEN‐deficient mice, BMI1+ luminal progenitor cells have been identified as the origin of tumors. Subsequent findings indicate that dead PCa cells, induced by ADT, transiently recruit T and B cell infiltration by releasing inflammatory factors, such as IL6, IL12, TNF‐α, and lymphotoxin.^[^ ^68^ ^]^ Furthermore, B cells recruited by IKKα and BMI1 produce lymphotoxin, which activates LTβR in PCa cells. This activation enhances the expression of IKKα, stimulating the NF‐kB and STAT3 pathways, ultimately leading to the development of CRPC. Moreover, the recruited B cells can express IgA, IL10, and PD‐L1, which are activated through the TGF‐β receptor pathway.^[^ ^69^ ^]^ These B cells may suppress the anti‐tumor function of CD8+ T cells in the PCa tumor microenvironment, thereby contributing to oxaliplatin resistance. In the context of BC, BMI1 is implicated in bladder tumorigenesis by diminishing the infiltration of CD8+ T cells.^[^ ^70^ ^]^ BMI1 promotes chemoresistance in BC.^[^ ^71^ ^]^ These evidence indicate that BMI1, which is regulated by AR, functions as an oncogene in both PCa and BC, contributing to chemotherapy resistance. Although there is currently no evidence to suggest that BMI1 regulates immune cells within the BC TME, we speculated, based on the PCa findings, that BMI1 likely plays a role in the regulation of immune cells in the BC TME. This presents a compelling hypothesis for investigating the function of BMI1 in immune regulation in BC. Furthermore, modulation of BMI1 expression may represent a shared target in both PCa and BC.

EZH2, a gene regulated by AR, is highly expressed in both mouse and human UGS during the early and intermediate stages of prostate development.^[^ ^72^ ^]^ The second peak of expression occurs at puberty.^[^ ^72^ ^]^ NK cells attracted by CRPC cells produce microRNA‐34 and microRNA‐449, which decrease transcription of the ARv7/EZH2 axis, consequently reducing the invasive ability of CRPC cells in vivo.^[^ ^73^ ^]^ GSK‐126, an EZH2 inhibitor, combined with ADT, restored the cytotoxic activity and enhanced IFN‐γ secretion of CD8+ T cells, effectively eliminating CRPC cells in vivo.^[^ ^74^ ^]^ In BC, EPZ011989, another EZH2 inhibitor, alone or in combination with cisplatin, significantly enhanced immune factors (such as IFN‐γ) in xenografts generated using HT1376 cells with KDM6A and SWI/SNF mutations.^[^ ^75^ ^]^ These factors stimulate natural killer (NK) cell activation, leading to tumor suppression. In a drug‐induced BC mouse model, EPZ011989 induces immune cell infiltration and activates antitumor functions (CD4+ and CD8+ T cells).^[^ ^76^ ^]^ Similarly, AR regulation in BC cells can influence PD‐L1 expression, thereby affecting the killing function of NK cells.^[^ ^77^ ^]^ Specifically, AR restored circ_0 001005 levels by suppressing the transcript of ADAR2 in BC cells. Restoration of circ_0 001005 promoted the sponging of miR‐200a‐3p, enhancing PD‐L1 expression. Ultimately, high PD‐L1 expression attenuates the killing function of NK cells, leading to immune escape from the TME, both in vitro and in vivo. EZH2 may suppress NK cell activation in both BC and PCa TME. Blocking EZH2 could be a promising strategy for improving the efficacy of chemotherapy by modulating the immune TME. Further studies are needed to explore the role of EZH2 in immunotherapy for the management of BC and PCa.

At E17, researchers knocked out YAP in stem cells of the UGS.^[^ ^78^ ^]^ YAP depletion hindered prostate regeneration and branching morphogenesis, which are mediated by the NOTCH and Hedgehog pathways during both fetal and maturation stages. YAP is a key gene in the Hippo pathway that is activated by the AR.^[^ ^79^ ^]^ Polymorphonuclear monocytic myeloid‐derived suppressor cells (MDSCs) have been identified as the primary infiltrating immune cells in PCa progression within the TME.^[^ ^80^ ^]^ Mechanistically, CXCL5 overexpression via the Hippo/YAP pathway activates CXCR2, thereby attracting polymorphonuclear MDSCs into the TME, which contributes to PCa progression. Blocking polymorphonuclear MDSCs effectively inhibits cancer progression in Pten/Smad4 mouse models. Similarly, PCa cells secrete platelet‐derived growth factor‐AA, which upregulates CXCL5 expression in bone marrow‐derived mesenchymal stromal cells by suppressing the MDA9/YAP/MST axis.^[^ ^81^ ^]^ CXCL5, in turn, facilitates PCa metastasis by promoting immunosuppressive TME. Moreover, YAP is also involved in the transition from the inflammatory phenotype of cancer‐associated fibroblasts (CAFs) to the myofibroblast phenotype via the mediation of the IL1α/ELF3/YAP pathway in PCa.^[^ ^82^ ^]^ However, few studies have investigated the role of YAP in bladder development. In BC, patients with TGF‐β and YAP/TAZ pathway inhibition may exhibit a higher response rate to immunotherapy.^[^ ^83^ ^]^ In both PCa and BC, YAP expression significantly correlates with immune evasion and promotes cancer progression. YAP and Hippo pathways are activated during prostate development and PCa. However, no studies have addressed the expression and function of YAP in bladder development. Furthermore, the correlation between the AR and YAP in BC remains unknown and requires further exploration in future studies.

FOXA1, which regulates AR expression in the prostate, is expressed in epithelial cells rather than stromal cells and is involved in prostate morphogenesis and differentiation during the embryonic phase.^[^ ^84^ ^]^ Similarly, FOXA1 is expressed throughout the entire process of bladder development, although its specific function requires clarification.^[^ ^41^ ^]^ The PAK4 knockout in PCa cells, a downstream gene of FOXA1, recruited CD8+ T cell infiltration and produced IFN‐γ, promoting hyperactivated angiogenesis and inducing a favorable TME in C57BL/6J mice.^[^ ^85^ ^]^ Additionally, PAK4 knockout enhanced the response rate of PCa cells to anti‐PD1 immunotherapy, thereby suppressing PCa progression. In BC cells, FOXA1 depletion increased PD‐L1 expression, as well as STAT2 and ASG15, by upregulating the transcription of IRF1 in an IFN‐ɣ‐independent manner.^[^ ^86^ ^]^ In another BC study, FOXA1 expression was negatively correlated with CD8 T cells and positively associated with M2 cancer‐associated macrophage (CAM) infiltration, as determined by the analysis of clinical samples and immunotherapy outcomes.^[^ ^87^ ^]^ Thus, FOXA1 expression contributes to the establishment of an immunosuppressive TME in both PCa and BC. In the prostate, FOXA1 regulates AR during the embryonic phase. However, to date, no study has explored the interaction between FOXA1 and the AR in immune modulation, which warrants further investigation.

KDM5B, a Jumonji C‐containing histone lysine demethylase, activates AR expression.^[^ ^88^ ^]^ KDM5B knockout in mice does not inhibit prostate development but promotes prostate hyperplasia and carcinogenesis.^[^ ^89^ ^]^ Based on the results of the PURE01 trial, researchers discovered that KDM5B diminishes immunogenicity in a specific subtype resistant to immunotherapy. KDM5B inhibition significantly enhances the immunogenicity of FGFR3‐mutated BC cells, potentially increasing the response rate to immunotherapy.^[^ ^90^ ^]^ During prostate development, PKM2 is primarily expressed in the prostate epithelial cells, whereas PKM1 is primarily expressed in the stromal cells.^[^ ^91^ ^]^ PKM2 plays an oncogenic role in PCa and is downstream of AR.^[^ ^92^ ^]^ In BC, PKM2 expression is reduced by the HNRNPL/circFAM13B/IGF2BP1 axis, disrupting PKM2‐induced immune evasion and creating a suppressive TME through metabolic reprogramming.^[^ ^93^ ^]^ KDM5B and PKM2 have been shown to promote prostate development and influence the immune microenvironment in BC. However, limited research has hindered a comprehensive discussion of the roles of KDM5B and PKM2 in the immune TME of PCa and BC.

The depletion of MAOA and MAOB in mice results in prostate epithelial atrophy, which primarily affects CK5 and P63 basal epithelial cells during development.^[^ ^94^ ^]^ The AR mediates MAOA transcription and influences the immune status of PCa.^[^ ^95^ ^]^ Another study on PCa showed that MAOA knockout in the TME led to increased infiltration of CD8+ T cells and elevated levels of immune factors, including granzyme B and IFN‐γ, in PTEN‐depleted mice.^[^ ^96^ ^]^ Furthermore, reduced MAOA levels can inhibit CAM polarization, thereby enhancing the response to immunotherapy.^[^ ^97^ ^]^ Similarly, AR can regulate immune factors, such as IFN‐γ, which influence the immune microenvironment. For instance, depletion of AR in CAFs restored levels of IFN‐γ and MCSF, consequently inhibiting cancer cell proliferation in vitro. AR expression in CD8+ T cells can also influence IFN‐γ expression by regulating the transcription of IFNG and GZMB, thereby inhibiting the function of CD8+ T cells in PCa.^[^ ^5^ ^]^ Inhibition of AR expression in CD8+ T cells may enhance the efficacy of PD‐1 targeted therapy through increased IFN‐γ expression. Immune factors play a crucial role in immune regulation. In another study, AR expression suppressed the transcription of IL8. Consequently, prostate epithelial cells undergoing ADT secrete IL8 (in humans) or CXCL15 (in mice) to activate the CXCR2 pathway, leading to the recruitment of polymorphonuclear MDSCs into the TME.^[^ ^44a^ ^]^ The combination of anti‐IL8 or anti‐CXCR2 with anti‐CTLA‐4 and ADT significantly enhanced PCa control by elevating the antitumor function of CD8+ T cells. In PCa, both granulocytic and monocytic MDSCs secrete reactive nitrogen species, such as nitrate lymphocyte‐specific protein tyrosine kinase in T cells, leading to a decline in T cell antitumor function in PTEN/P53/SMAD4 mouse models.^[^ ^98^ ^]^ IL1β, a subtype of IL1, also affected the PCa immune TME.^[^ ^99^ ^]^ Specifically, ADT enhances IL1β expression by suppressing AR function in MDSCs, which in turn attenuates the antitumor function of CD8+ T cells. Inhibition of T cells results in decreased IL2 secretion, inducing an immunosuppressive TME. Reactive nitrogen species‐reducing agents could significantly enhance the antitumor efficacy of immunotherapy, such as anti‐PD1 or anti‐CTLA4. In the context of BC, coculture with B cells promotes BC cell progression by modulating IL8, AR, and MMPs both in vitro and in vivo.^[^ ^100^ ^]^ Furthermore, BC cells recruit CD4+ T cells, which enhance their invasive and metastatic potential. Specifically, these recruited CD4+ T cells facilitate BC progression through the regulation of the IL1, IL8, AR, HIF1α, and VEGFa pathways.^[^ ^101^ ^]^ Genes such as MAOA not only influence the development of the prostate and bladder during embryonic stages but also regulate tumor immunity through immune factors and AR expression, thereby affecting tumor progression and treatment. Disrupting or enhancing specific immune factors within the TME may provide a strategy for simultaneously controlling shared pathways in tumor biology. This approach can rectify immune disruptions caused by the AR or other factors, ultimately preventing tumor progression and improving treatment outcomes.

AR promotes BC and PCa. However, recent studies have revealed that the AR exhibits distinct functions in regulating immune responses across different cell types, a characteristic that is evident during development. This raises the question of whether the roles of AR are consistent across all BC cell types. Can AR‐mediated immune regulatory outcomes vary in different cell types? In clinical studies, the effect of AR expression and ADT on BC remains controversial, although most studies suggest that ADT can improve the prognosis of BC.^[^ ^102^ ^]^ The differential response of BC cells to AR expression and ADT treatment is primarily attributed to the varying roles of AR expression in different cell types, which may lead to contradictory results. In PCa, AR inhibition in different cell types, such as epithelial and stromal cells, results in changes in the immune response and inflammation within the TME, ultimately affecting cancer progression and treatment efficacy.^[^ ^53^ ^]^ These cell types exhibit different androgen and AR requirements during prostate development, which may explain this phenomenon. This suggests that to resolve the contradictory findings regarding AR in BC and to advance the recent positive outcomes of antiandrogen combined immunotherapy, it is essential to understand the role of AR in bladder development, to enhance our understanding of its specific function in immune regulation in BC.^[^ ^103^ ^]^

### 3.3 The Progression of PCa and BC with AR‐Related Pathways

Many pathways are involved in the development of the bladder and prostate and affect the progression of BC and PCa by modulating the immune microenvironment. Of these, TGF‐β is involved in prostate and bladder development.^[^ ^104^ ^]^ During the embryonic stages E12.5 to E16.5, BMP4, and TGF‐β1 are expressed in the transitional epithelium, lamina propria, and muscularis mucosa.^[^ ^105^ ^]^ Thus, AR may mediate mesenchymal and epithelial interactions by regulating the TGF‐β pathway.^[^ ^106^ ^]^ In bladder development, TGF‐β knockout leads to hypertrophy of the lamina propria and muscularis externa, accompanied by myofibroblast differentiation and proliferation in male mice. However, TGF‐β depletion does not affect bladder development in female mice due to the modulation of estrogens and androgens.^[^ ^107^ ^]^ In PCa, ADT recruits B cell infiltration by CXCL13+ myofibroblasts, promoting the development of CRPC.^[^ ^108^ ^]^ Mechanistically, hypoxia‐induced activation of HIF1α enhances the generation of CXCL13+ myofibroblasts by upregulating the TGF‐β pathway. In mouse models of bone metastasis, TGF‐β released by CRPC cells induces the development of Th17 cells instead of Th1 cells, thereby diminishing the effectiveness of immunotherapy.^[^ ^12^ ^]^ Blocking TGF‐β enhances the presence of Th1 cells and CD8+ T cells, thereby controlling CRPC progression. In addition to cancer cells producing TGF‐β, T cells also release TGF‐β1, which negatively impacts antigen‐specific T cell responses. Notably, blocking T cell‐derived TGF‐β1 is sufficient to control PCa progression.^[^ ^109^ ^]^ Furthermore, TGF‐β receptor II (TGF‐βRII) reduces the efficacy of CAR T‐cell therapy in PCa, whereas TGF‐βRII depletion in CAR T‐cells significantly enhances their antitumor function in vivo.^[^ ^110^ ^]^ BC cells facilitated CAM M2 polarization by activating the HIF1α/TGF‐β pathway.^[^ ^111^ ^]^ M2 CAMs increase PD‐L1 expression in BC cells through the TGF‐β/Smad2/3/METTL3 pathway, contributing to an immune‐evading TME. Additionally, TGF‐β and IL10, activated by S1P1 in BC cells, facilitated the infiltration of Tregs. In contrast, inhibiting TGF‐β or IL10 significantly suppresses this recruitment.^[^ ^112^ ^]^ Thus, TGF‐β inhibitor is a promising drug for enhancing immunotherapy efficacy in BC.^[^ ^113^ ^]^ The evidence suggests that the TGF‐β pathway plays a critical role in bladder and prostate development, while also being involved in the progression of BC and PCa. In cancer cells, regulation of the TGF‐β pathway can induce either an antitumor or pro‐tumor immune microenvironment in local or metastatic TMEs. Furthermore, directly targeting the TGF‐β pathway in immune cells can markedly enhance their anticancer functions. These findings indicate that the TGF‐β pathway may serve as a promising target for controlling BC and PCa through immune modulation.

The WNT/β‐catenin pathway, regulated by AR, is activated and plays a crucial role during prostate development.^[^ ^114^ ^]^ SOX9 promotes the emergence of prostate stem cells by upregulating the WNT/β‐catenin pathway.^[^ ^115^ ^]^ Expressed in basal epithelial cells, SOX9 mediates AR expression and facilitates prostate development; however, SOX9 knockout prior to AR activation can inhibit prostate initiation.^[^ ^116^ ^]^ During bladder development, SOX9 primarily influences epithelial cell proliferation and differentiation.^[^ ^117^ ^]^ SOX9 promotes bladder carcinogenesis by modulating the EGFR/ERK/SOX9 pathway.^[^ ^117b^ ^]^ In BC, non‐T cell inflamed tumors are associated with hyperactivated β‐Catenin, PPARγ, and FGFR3 pathways, which contribute to the formation of an immune‐suppressive TME.^[^ ^118^ ^]^ The AR/WNT/β‐catenin pathway also modulates prostate development through the BMP pathway.^[^ ^119^ ^]^ GALNT12 hyperactivates BMPR1A expression, inhibiting the STAT3 pathway, which suppresses M2 macrophage infiltration and promotes DC and NK cell infiltration in vivo.^[^ ^120^ ^]^ In contrast, in BC, BMP4 produced by BC cells stimulates M2 CAM polarization.^[^ ^121^ ^]^ Furthermore, miRNA21 suppresses BMPR2 expression, thereby neutralizing the antitumor effects of BMP4. The WNT/β‐catenin pathway and its associated network are involved in the regulation of the immune microenvironment, subsequently affecting the progression of BC and PCa. Given the controversial results concerning the BMP pathway, specific function of a gene within a pathway may vary across different cancers. This phenomenon may also be related to AR and androgen expression.

WNT and SHH pathways play significant roles in bladder development. Dysregulated expression of these two pathways can induce bladder dysplasia.^[^ ^122^ ^]^ AR negatively regulates the SHH pathway.^[^ ^123^ ^]^ AR knockout in stromal cells activates the SHH pathway, which in turn leads to the failure of wild‐type prostatic epithelial cell‐mediated development of normal prostatic epithelia during puberty and disrupts prostatic budding during embryogenesis. In PCa, KLF4, a gene downstream of GLI1, may promote cancer progression by attenuating CD8+ T cell infiltration and suppressing CAM M2 polarization.^[^ ^124^ ^]^ In BC, SHH is negatively correlated with carcinogenesis; basal cells expressing SHH serve as precursor lesions that develop into tumor‐initiating cells but disappear in muscle‐invasive BC.^[^ ^125^ ^]^ However, some studies have reported that the SHH pathway promotes BC progression and chemoresistance.^[^ ^126^ ^]^ Although its role remains debatable, the SHH pathway is essential for the development of both the bladder and prostate. This phenomenon may be analogous to previous findings suggesting the varying immunomodulatory effects of AR in different cellular contexts. The pathways regulated by AR at various stages and cell types may also exhibit differential immunoregulatory effects.

These results suggest that the genes and pathways activated during development are also frequently expressed in cancer or stromal cells, indicating that the active regulatory networks of development and cancer are comparable. This network may create an immune microenvironment that influences tumorigenesis, progression, and treatment efficacy in BC and PCa. However, in checkpoint immunotherapy, patients with BC and PCa exhibit markedly different outcomes. This discrepancy may stem from the presence of one or more regulatory genes or pathways. Researchers have typically focused on the differences between normal and cancerous tissues to uncover the underlying causes of immune variation. However, it is crucial to acknowledge the significant heterogeneity of cancer and cancer‐associated stromal cells across various disease stages, patient populations, cell lines, and animal models. Consequently, identifying the key factors contributing to immune differences between BC and PCa from a tumor‐centric perspective is highly challenging and susceptible to substantial heterogeneity bias. Many genes and pathways are expressed at both developmental stages and in cancer tissues. Rapid growth, metabolic activation, and stem‐like characteristics are hallmarks of this developmental stage and have also been observed in cancer tissues. The primary distinction between developmental stages and cancer lies in the controllability of cell growth and divergent trajectories of stem cell differentiation. Therefore, understanding and comparing key genes and pathways involved in bladder and prostate development may provide valuable insights into the immune differences between BC and PCa. Furthermore, identifying and elucidating critical nodal genes involved in developmental processes, such as those controlling growth and differentiation, could significantly enhance and direct tumor research. Notably, the AR and androgens represent major differences between bladder and prostate cancers, offering a promising avenue for investigating immune disparities between BC and PCa **Figure** **2** exhibited above regulatory mechanism.

### 3.4 The Immune Regulation of Androgen/AR and Treatment in PCa and BC

ADT effectively controls PCa cells by blocking the AR signaling pathway. AR knockout in luminal cells induces prostate inflammation.^[^ ^127^ ^]^ Similarly, ADT changes the immune microenvironment of PCa and is thus a promising way to improve the efficiency of immunotherapy.^[^ ^128^ ^]^ For instance, a clinical study reported that patients with PCa undergoing ADT exhibited increased infiltration of CD4+ and CD8+ T cells.^[^ ^129^ ^]^ The presence of AR in CD8+ T cells can modulate IFN‐γ expression by regulating the transcription of IFNG and GZMB, thereby inhibiting the function of CD8+ T cells.^[^ ^5^ ^]^ Consequently, ADT may enhance the antitumor function of CD8+ T cells by reducing AR expression. However, the KEYNOTE‐641 trial demonstrated that the combination of pembrolizumab, enzalutamide, and ADT did not yield improved survival outcomes compared to enzalutamide plus ADT.^[^ ^130^ ^]^ This indicated that patients with PCa did not benefit from the combination of immunotherapy and ADT, which contrasts with the findings of previous studies. Subsequent investigations revealed that several factors contribute to this lack of benefit. ADT induces cytokine production and recruits immune cells, resulting in an immune‐suppressive TME.^[^ ^131^ ^]^ For example, CAMs significantly affect the efficacies of ADT and immunotherapy.^[^ ^132^ ^]^ AR knockdown in PCa recruits CAMs by activating CCL2‐induced CCR2/STAT3 and epithelial‐mesenchymal transition (EMT) pathways, thereby promoting PCa progression.^[^ ^133^ ^]^ In addition, CCL2 secreted by stem cells facilitates monocyte and CAM infiltration in PCa.^[^ ^134^ ^]^ CCL2 produced by PCa cells also recruits M2 CAMs and induces Treg infiltration.^[^ ^135^ ^]^ In mouse models and human tissues, ADT treatment induces CD163+ (M2 CAMs selective marker) CAM infiltration.^[^ ^136^ ^]^ Specifically, PCa cells treated with ADT secrete macrophage‐recruiting cytokines, including CCL2 (MCP‐1), SDF1, IL34, and CSF1, to attract pro‐tumor CAMs. Furthermore, the use of anti‐CSF1/CSF1R agents has demonstrated synergistic effects with ADT in managing PCa by reducing pro‐tumor CAM infiltration both in vitro and in vivo. A randomized controlled trial (NCT01696877) showed that ADT could recruit CD8+ T cells and induce regulatory T (Treg) cell infiltration.^[^ ^137^ ^]^ Patients receiving ADT in combination with a GM‐CSF‐secreting allogeneic cellular vaccine and cyclophosphamide exhibited better clinical outcomes than those receiving ADT alone, suggesting that Treg‐depleting agents represent a promising strategy for enhancing the efficacy of ADT. Moreover, CHIT1, a macrophage marker, was upregulated in the combination group, which has been proposed as a potential explanation for immunotherapy resistance in PCa.^[^ ^138^ ^]^ In women, FOXP3 can be enhanced by the AR both in vitro and in vivo, attracting Treg infiltration in vitro.^[^ ^139^ ^]^ These findings may partially explain why ADT‐induced T cell infiltration does not improve survival outcomes in patients with PCa.^[^ ^140^ ^]^

Immune cell function is a critical factor in treatment failure. For instance, a study observed that ADT using leuprolide and flutamide can influence the antitumor function of T cells by regulating GABAA‐induced IFN‐γ expression.^[^ ^141^ ^]^ Consequently, abiraterone, a drug that does not target GABAA receptors and can suppress 3βHSD and AR, effectively enhances the efficacy of immunotherapy. This may explain the poor results observed with immunotherapy in combination with enzalutamide and ADT (NCT03834493). Therefore, targeting AR may represent a promising approach for controlling PCa by regulating immune cell function. For example, researchers developed a DNA vaccine targeting the AR ligand‐binding domain (pTVG‐AR) that induces a TH1‐type immune response to eliminate PCa cells.^[^ ^142^ ^]^ In the subsequent trial (NCT02411786),^[^ ^143^ ^]^ patients with pTVG‐AR showed continued IFN‐γ immune responses. Patients with a TH1‐type immune response demonstrated improved 18‐month PSA progression‐free survival with minimal side effects. These findings suggest that the efficacy of immunotherapy in PCa can be enhanced by modulating AR expression in both tumor and immune cells. Moreover, they underscore the importance of precision medicine in this process. Tailoring AR modulation strategies to target specific cells based on individual TME analyses may enhance patient survival benefits from immunotherapeutic agents.

In a Phase 2 clinical trial (NCT03951831), the combination of ADT and anti‐PD1 treatment resulted in a significant increase in CD8+ T cell infiltration in metastatic samples from the bone, liver, and lungs, which was not observed in the ADT‐only group.^[^ ^128^ ^]^ Notably, in the lymph nodes, ADT combined with anti‐PD1 treatment led to a slight increase in immune cell infiltration, characterized by a higher proportion of regulatory Tregs and myeloid cells. In contrast, the proportion of tumor cells was higher in the ADT + anti‐PD1 group than in the ADT group. This study presents an alternative perspective indicating that metastatic PCa cells from various sites exhibit distinct AR and immune profiles. This variation may be attributed to the diverse origins of metastatic cancer cells in the TME. Thus, consistent with our previous speculation, the relationship between AR and immunity appears to depend on the cell source. Therefore, elucidating the association between the AR and relevant genes or pathways involved in prostate development could facilitate the investigation of the interplay between the AR and immunity in PCa cells originating from different sources.

**Figure 3:**
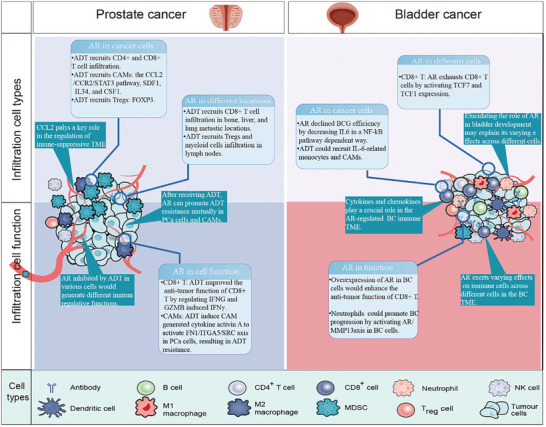
The role of androgens and androgen receptors (AR) in the treatment of prostate and bladder cancer: AR can impair the effectiveness of drug treatments by affecting immune cell infiltration and immune cell function; AR exhibits different immunomodulatory functions in different cells. AR: androgen receptor; BCG: Bacillus Calmette–Guerin; CAMs: cancer‐related macrophages; BC: bladder cancer; ADT: androgen ablative therapy; PCa: prostate cancer; TME: tumor microenvironment.

BCG is the standard immunotherapy for patients with moderate‐ and high‐risk NMIBC.^[^ ^144^ ^]^ AR overexpression has been shown to decrease IL‐6 levels by attenuating the NF‐kB pathway in BC cells, subsequently reducing the efficacy of BCG therapy.^[^ ^145^ ^]^ Specifically, AR expression diminishes the effectiveness of BCG; however, the combination of BCG with ADT or the AR degradation enhancer ASC‐J9 enhances BCG efficacy by recruiting IL‐6‐related monocytes and CAMs in mouse models.^[^ ^146^ ^]^ Mizushima et al.^[^ ^15^ ^]^ reported that the AR decreased the efficacy of BCG by enhancing RAB27B/SYTL3‐induced exocytosis. Another study indicated that combining BCG or anti‐PD‐L1 therapy with ADT enhanced the efficacy of immunotherapy in vivo.^[^ ^103^ ^]^ BC patients who received 5α‐reductase inhibitors had fewer cancer‐specific deaths than those who did not.^[^ ^147^ ^]^ The AR‐mediated reduction in TCF7 and TCF1 expression in CD8+ T cells may lead to T cell exhaustion, contributing to an immunosuppressive TME and reduced immunotherapy efficacy in BC.^[^ ^44b^ ^]^ Furthermore, neutrophils have been implicated in the AR‐induced progression of BC. Specifically, neutrophils recruited by BC cells can activate AR expression, enhance MMP13 transcription in vitro, and promote BC progression.^[^ ^148^ ^]^ In BC, additional findings revealed that the AR inhibits PD‐L1 expression by binding to the promoter region of PD‐L1. In C3H/HeN mouse models, AR overexpression in BC cells significantly increases the efficacy of anti‐PD‐L1 therapy by promoting CD8+ T cell infiltration, although AR overexpression alone does not affect CD8+ T cell infiltration.^[^ ^4^ ^]^ Further results indicated that AR overexpression in BC cells cocultured with CD8+ T cells could significantly improve the antitumor function of CD8+ T cells, thus enhancing the efficiency of immunotherapy.^[^ ^4^ ^]^ These studies highlighted the diverse effects of AR expression in different cell types. Similar to PCa, modulating the AR in BC can simultaneously influence immune cell infiltration and functionality. Changes in AR expression in various cell types result in distinct immune responses. Consequently, elucidating the relationship between the AR and immunity in different cell types within BC, as well as investigating the role of the AR in bladder development, may enhance our ability to manage tumor occurrence, progression, and treatment through immune modulation. **Figure** **3** illustrates the role of the AR in the treatment of PCa and BC, and **Table** **1** presents key references regarding estrogen receptor (ER) involvement in the development of PCa and BC.

**Table 1:** The key references of androgen and androgen receptor in prostate cancer and bladder cancer.

PMID	Organ	Stage	Cell line	Animal	Pathway/gene
27 594 448	Prostate	Carcinogenesis	Luminal cell	K8‐AR Mice	IL1
37 061 215	Prostate	Carcinogenesis	BPH‐1	Male Sprague‐Dawley rat	The AR/TGF‐β/NOX4 pathway
19 844 236	Prostate	Carcinogenesis	/	Male C3H/HeOuJ mice	/
22 010 203	Prostate	Carcinogenesis	/	HFD rabbit	/
22 745 041	Prostate	Carcinogenesis	Fibromuscular cell	C57BL/6 mice	The NF‐kB pathway
15 766 662	Prostate	Carcinogenesis	T cell	TRAMP mice	/
22 086 872	Bladder	Carcinogenesis	SVHUC, 293T	Male ARKO mice	UDP‐glucuronosyltransferases.
34 400 221	Bladder	Carcinogenesis	293T, T24, HT‐1197, 639V, 647V, MDA‐MB‐468, and U2OS	Mice	RNF144A upregulated DNAPKcs, BMI1, and PDL1.
29 402 932	Prostate	Progression	LNCaP, VCaP, C4‐2, and 22Rv1	CB17SCID mice	BMI1 and AR complex.
20 220 849	Prostate	Progression	Myc‐CaP	BL6 mice	IKKβ/IKKα/LTβR/NF‐kB and IKKβ/LTβR/STAT3 pathway.
25 924 065	Prostate	Progression	/	C57BL/6 and FVB mice	IKKα/BMI1/TGFβ pathway activates IgA, IL10 and PD‐L1.
23 038 103	Prostate	Development	LNCaP, C4‐2B, and PC‐3	C57B6/J breeder pairs	ANCCA/EZH2.
28 373 004	Prostate	Progression	NK92, CWR22Rv1, and VCaP	Nude mice	microRNA‐34 and microRNA‐449 activated ARv7/EZH2 axis.
30 692 641	Bladder	Progression	HT1376, T24, and UM‐UC‐3	Nude mice	EZH2.
36 197 969	Bladder	Progression	/	C57BL/6 mice	EZH2.
37 689 711	Prostate	Development	/	C57BL/6 mice	YAP activates Notch and Hedgehog pathways.
37 385 752	Prostate	Progression	HEK293T, LNCaP and C4‐2	/	AR/YAP/TAZ pathway.
26 701 088	Prostate	Progression	Ptenpc and Smad4pc depletion prostate cell	C57BL/6 mice	Hippo‐YAP pathway/CXCL5‐CXCR2 axis.
37 922 327	Prostate	Progression	HS5, PC‐3ML‐Luc, RM1, RM1‐BM	Mice	PDGFAA/CXCL5/YAP/MST pathway and MDA9/CXCL5 axis.
32 182 655	Bladder	Progression	/	/	TGF‐β and YAP/TAZ pathways.
36 509 363	Prostate	Progression	RM1, CRL‐2505 and LNCaP	C57BL/6J mice	FOXA1/AR/PAK4 axis.
36 564 410	Bladder	Progression	UMUC1 and UMUC3	/	FOXA1/IRF1 aixs suppressed PD‐L1, STAT2, and ASG15 expression.
34 706 362	Bladder	Progression	/	/	FOXA1.
33 245 716	Prostate	Development	/	Male PB‐Cre4 mice	KDM5B.
37 105 962	Bladder	Progression	RT4	/	KDM5B.
38 680 860	Prostate	Development	VCap, LNCaP, C4‐2B, 22RV1, PC3, and DU145	/	PKM1 an PKM2.
36 747 239	Bladder	Progression	SV‐HUC, BIU87, T24, RT4, 5637, 253 J, UMUC3, and J82	NOG (NOD‐scid IL2Rg null) mice	HNRNPL/circFAM13B//IGF2BP1/PKM2 pathway.
29 637 670	Prostate	Development	RWPE‐1	Wild‐type and MAOA/B‐knockout mouse	MAOA and MAOB.
36 242 915	Prostate	Progression	/	MAO A/Pten DKO mice and C57 B/L male mice	MAOA.
32 520 720	Prostate	Development	/	Male Wistar rats	ERK1/2 and Wnt5a.
23 620 745	Bladder	Development	/	Normal, timed mated outbreed CD1 mice	TβR1 and BMP4.
25 267 627	Prostate	Progression	/	TRAMP (Bl6×129) mice	HIF1 and TGF‐β pathway induced CXCL13 secretion.
31 730 856	Bladder	Progression	Myc‐CaP	Male FVB/NJ mice	TGF‐β pathway.
21 757 379	Prostate	Progression	B16 and EL‐4	TRAMP mice	TGF‐β pathway.
37 690 238	Prostate	Progression	PC3 and LNCAP	Female NOD/SCID IL‐2gamma KO (NSG) mice	TGF‐βRII and TIM3.
30 718 502	Prostate	Progression	EJ, T24, Biu87, and J82	/	S1P1 activated the TGF‐β pathway and IL10 secretion.
36 906 247	Bladder	Development	/	zebrafish	The Wnt/β‐catenin pathway.
23 087 175	Prostate	Development	/	C57BL/6J mice	WIF1.
22 761 195	Prostate	Development	/	TRAMP mice	SOX9.
17 234 760	Prostate	Progression	LNCaP, CWR22, PC3, and DU145	/	SOX9/AR axis.
25 127 126	Bladder	Development	/	Mice	SOX9, SMD4, TBX18, and SMAD4.
27 197 067	Bladder	Progression	/	/	β‐Catenin, PPARγ, and FGFR3 pathways.
23 396 188	Prostate	Development	/	Mice	β‐Catenin/BMP pathway.
28 928 159	Bladder	Progression	T2, 5637, J82, 97‐1, 97‐7, MGH‐U3, MGH‐U4, RT112, UMUC1, UMUC5, UMUC7, and VMCUB1.	/	BMP4, miR21/BMPR2 pathway.
37 478 986	Bladder	Development	/	Zebrafish.	Wnt/Hedgehog pathway.
35 490 551	Bladder	Development	/	Zebrafish.	Wnt/Hedgehog pathway.
34 427 305	Prostate	Development	/	Mice.	AR/SHH pathway.
29 324 844	Prostate	Progression	B6 Hi‐Myc	Wild‐type C57BL/6 mice.	KLF4.
25 314 078	Bladder	Progression	ScienCell 4320, ScienCell 4330, ScienCell 4321, and ScienCell 2301.	Male mice.	SHH and BMP pathways.
33 552 265	Bladder	Progression	UMUC3 and EJ.	/	SHH pathway.
35 167 374	Bladder	Progression	T24 and EJ.	Female BALB/c nude mice	BRD4/SHH pathway.
35 322 234	Prostate	Treatment	/	C57BL/6, RIP‐mOVA, OTI, C57Bl/6; CD90.1 congenic mice.	AR/IFNG and AR/GZMB induced IFN‐γ.
37 922 910	Prostate	Treatment	/	/	TNF‐α.
11 734 652	Prostate	Treatment	/	/	IFN‐γ.
36 749 798	Prostate	Treatment	MycCaP‐Bo.	FVB/N mice.	Cytokine activin A activates FN1/ITGA5/SRC axis
23 982 944	Prostate	Treatment	RAW264.7, C4‐2, TRAMP‐C1, THP‐1, LNCaP, and LAPC4.	MARKO/TRAMP C57BL/6 Mice.	AR/CCL2/CCR2‐STAT3 axis.
31 327 655	Prostate	Treatment	PC3M, 293FT, LNCaP, 22rv1, VCaP, DU145, PC3, Myc‐Cap, TRAMP‐C1, and PC12.	Male BALB/c nude mice, male NOD SCID gamma mice, male C57BL/6J mice, and male FVB/NJ mice.	CCL2.
25 736 687	Prostate	Treatment	RAW264.7, Myc‐CaP, LNCAP, LNCaP‐C4‐2, and CWR22Rv1.	FVB male mice.	CSF1.
32 173 650	Prostate	Treatment	UV‐8101‐RE and TRAMP‐C1.	C57BL/6 mice.	IL2.
28 321 130	Prostate	Treatment	mES.	PB‐Cre+ PtenL/L p53L/L Smad4L/L mice.	IL1.
27 053 771	Prostate	Treatment	Myc‐CaP, MC38, and B16‐EGFR.	FVB male mice and C57BL/6 OTI and OTII mice.	IFN‐γ.
23 108 626	Prostate	Treatment	A2/TRAMP.	HHDII‐DR1 transgenic mice, C57Bl/6 mice.	AR.
14 532 843	Bladder	Treatment	253J and T24.	/	AR, IL6 and the NF‐kB pathway.
37 059 268	Bladder	Treatment	/	C3H/HeN mice.	AR and PDL1.
26 264 279	Bladder	Treatment	T24, 253J, and MB49.	FVB female mouse.	IL6, TNF‐α and AR
26 517 808	Bladder	Treatment	T24, J82, and SV‐HUC‐1.	/	AR/MMP13 axis.

## 4 Comparing Estrogen and ER Between PCa and BC: An Immunological Perspective

### 4.1 The Tumorigenesis of Prostate and Bladder

ER includes the ER‐α and ER‐β subtypes.^[^ ^149^ ^]^ ER‐α is expressed in 0%–38% of BC samples, while ER‐β is expressed in 27%–100% of BC tissues.^[^ ^150^ ^]^ High estrogen levels can induce squamous metaplasia in the prostatic utricle, whereas this phenomenon are not observed in the bladder.^[^ ^151^ ^]^ ERα is typically expressed in prostate basal epithelial cells and stromal cells, while ERβ is primarily found in prostate epithelial cells, particularly in luminal cells.^[^ ^152^ ^]^ The Erα knockout in smooth muscle cells leads to a decline in glandular infolding in the proximal section by downregulating IGF1 and EGFR, while depletion of ERα reduces the number of ductal tips.^[^ ^153^ ^]^ ERα primarily promotes AKT expression, whereas ERβ selectively activates MAPK cascades in the prostate.^[^ ^154^ ^]^ During prostate cancer development, β‐catenin promotes CCND1 expression. Subsequently, estrogen may improve CCND1 expression.^[^ ^21^, ^155^ ^]^ ERα acts as a promotor in prostate carcinogenesis, while ERβ or ERβ1 serves as a protector against PCa occurrence.^[^ ^156^ ^]^ Both ERα and ERβ involve in bladder carcinogenesis.^[^ ^150^ ^]^ Further studies indicate that ERα inhibits tumorigenesis, while ERβ promotes cancer initiation in vivo.^[^ ^157^ ^]^ Schistosoma haematobium infection in the bladder can induce carcinogenesis, often characterized by squamous differentiation, primarily driven by chronic inflammation.^[^ ^158^ ^]^ In addition to nuclear receptors, membrane sex hormone receptors, including ER, also contribute to disease occurrence and progression.^[^ ^64^, ^159^ ^]^ For instance, GPR30, a type of GPER located in the endoplasmic reticulum, can be activated by estrogen to enhance intracellular calcium mobilization and generation of phosphatidylinositol 3,4,5‐trisphosphate, thereby influencing disease development and progression.^[^ ^159^ ^]^ In the prostate, YAP overexpression has been observed in benign prostatic hyperplasia and is associated with lower levels of estrogen and GPER expression.^[^ ^160^ ^]^ GPER upregulation results in YAP phosphorylation and degradation, inhibits prostatic epithelial proliferation by enhancing apoptosis and may serve as a strategy to prevent prostate carcinoma. Given the sex‐based differences in ER expression, female and male BC patients exhibit distinct gene expression profiles and immune microenvironments, leading to poorer survival outcomes for female patients than for their male counterparts.^[^ ^2^, ^161^ ^]^ Similar to AR, ER can also induce inflammation to promote prostate carcinogenesis. However, there are notable differences in the roles of ER subtypes in prostate and bladder carcinogenesis. ERα can suppress tumorigenesis in the bladder while promoting the development of PCa. In contrast, ERβ plays an opposite role to ERα in bladder and prostate carcinogenesis. This contrasting role presents challenges in the selection of estrogen‐related foods and drugs. Furthermore, limited research has addressed the involvement of ERα and ERβ in the development of the bladder and prostate, hindering our understanding of these distinct functions.

### 4.2 The Immune Modulation in PCa and BC Progression

In PCa studies, researchers typically categorize ERβ into five subtypes: ERβ1 (the full‐length form, commonly referred to as ERβ), ERβ2, ERβ3, ERβ4, and ERβ5.^[^ ^156b^ ^]^ Similarly, ERα is positively correlated with PCa progression.^[^ ^162^ ^]^ In BC progression, estrogens and ERβ facilitate cancer development both in vitro and in vivo, while the role of ERα in this process remains ambiguous.^[^ ^163^ ^]^ Estrogen and its receptors regulate inflammatory and immune responses by modulating macrophages and monocytes.^[^ ^164^ ^]^ ERα expression in RAW 264.7 CAMs has been shown to suppress macrophage‐related inflammation by modulating endotoxin‐induced MMP9 expression and morphology.^[^ ^164^, ^165^ ^]^ Coculture with mast cells, BC cells had higher invasion ability by activating the ERβ/CCL2/CCR2/EMT/MMP9 pathway in vitro and in vivo.^[^ ^166^ ^]^ Estrogen activates MMP2 expression, leading to the generation of TGF‐β1 through targeting ERα in prostatic stromal cells (WPMY‐1).^[^ ^167^ ^]^ Subsequently, TGF‐β1 stimulates MMP2 expression in PCa cells, contributing to cancer progression. As previously mentioned, TGF‐β1 is involved in the development of both the prostate and bladder. ERα influences TGF‐β1 to promote PCa progression by mediating MMP2, a member of the MMP family. Additionally, in BC, ERβ enhances the invasive ability of cancer cells by influencing MMP9 expression. These findings present two significant insights: first, in alignment with carcinogenesis, ERα facilitates the advancement of PCa, whereas ERβ supports cancer progression in BC. Second, by modulating the MMP family, ERs may play a critical role in regulating cancer progression. However, there is a paucity of research regarding the role of the MMP family in bladder and prostate development, indicating the need for further investigation in future studies.

Modulation of immune factors represents a significant pathway through which ER influences tumor immunity and progression. For example, CCL5 has been implicated in PCa chemoresistance by affecting T cells.^[^ ^168^ ^]^ Specifically, CD4+ T cells produce CCL5, which enhances the STAT3 pathway in vivo, contributing to docetaxel resistance in PCa. Additionally, CAFs stimulated by ERα can secrete CCL5, which attenuates M2 macrophage infiltration and reduces IL‐6 expression in M2 macrophages, thereby inhibiting PCa progression both in vitro and in vivo.^[^ ^169^ ^]^ Moreover, BMP6 secreted by PCa cells induces IL‐6 expression in macrophages, promoting neuroendocrine differentiation of PCa.^[^ ^170^ ^]^ In the context of BC, ERα in CAFs enhances the transcription of CCL1 in fibroblasts and promotes IL‐6 secretion in BC cells, facilitating BC invasion.^[^ ^171^ ^]^ Similar to PCa, CCL2 in BC cells can also attract CAM infiltration, facilitating the lymphatic metastasis of BC.^[^ ^172^ ^]^ Furthermore, CD4+ T cells recruited by BC cells can enhance metastatic potential by activating the ERβ/IL‐1/c‐MET pathway in vitro and in vivo, an effect that can be partially reversed by IL‐1 or c‐MET inhibitors.^[^ ^173^ ^]^ Finally, EBAG9, an estrogen‐responsive gene, may influence BC progression by regulating immune cell infiltration into the TME.^[^ ^174^ ^]^ Mechanistically, the BC TME in mice with EBAG9 knockout exhibited increased infiltration of CD8+, CD3+, and CD4+ T cells, enhancing the antitumor function of CD8+ T cells compared to the control group, which resulted in the suppression of BC cells. Interestingly, in the prostate, ERα expression in CAFs can inhibit IL6 expression, thereby suppressing the formation of an immunosuppressive environment and controlling tumor progression. However, ERα expression in PCa cells promotes tumor progression.^[^ ^162^ ^]^ In BC, ERα expression in CAFs promotes BC progression. However, ERα expression in BC cells inhibits BC progression.^[^ ^175^ ^]^ Therefore, we hypothesized that different ER subtypes exhibit distinct functions in various cell types, particularly cancer and stromal cells. This observation is analogous to previous findings regarding AR, where its relationship with immune regulation varied across different cell types.^[176]^ Furthermore, few studies have investigated the roles of other ERβ subtypes, including ERβ2‐5, in immune modulation. One study has identified that ERβ2 regulates immune function in fish macrophages.^[^ ^177^ ^]^ The immune regulatory roles of ERβ3‐5 remain unclear, although some studies have identified their functions in cancer occurrence and progression.^[^ ^178^ ^]^ Thus, we strongly advocate that future research should focus on elucidating the roles of different ER subtypes in various cell types during both normal and cancer development.

### 4.3 The Immune Regulation of Estrogen/ER and Treatment in PCa and BC

**Figure 4:**
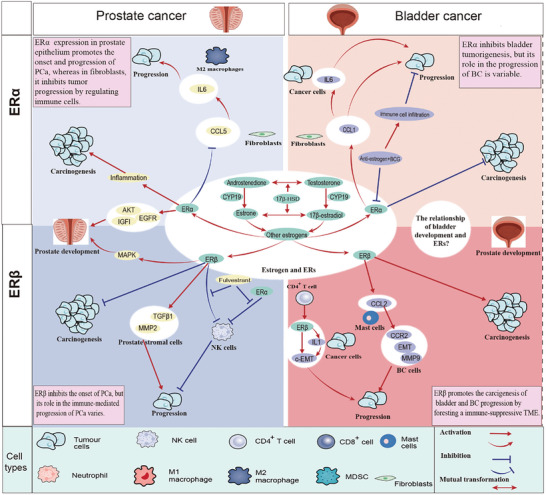
The role of estrogens and estrogen receptors (ER) in the development, carcinogenesis, and progression of prostate and bladder cancer: ERα and ERβ have distinctly different roles in prostate and bladder cancers; modulating ER expression in bladder and prostate cancers can significantly alter the efficacy of immunotherapy; ERs exhibit different immunoregulatory functions in various cells. 17β‐HSD: 17β‐hydroxysteroid dehydrogenase; BC: bladder cancer; PCa: prostate cancer; ER: estrogen receptors; BCG: Bacillus Calmette–Guerin.

The estrogen/ER ratio affects the efficacy of immunotherapy.^[^ ^179^ ^]^ A study on breast and colorectal cancer demonstrated that the overexpression of ERβ has a synergistic effect with anti‐PD1 therapy.^[^ ^180^ ^]^ The administration of an ERβ agonist increased the infiltration of CD8+ T cells while attenuating the levels of CSF1 from tumors, subsequently reducing the population of CSF1R+ myeloid‐derived suppressor cells and enhancing the effectiveness of anti‐PD1 therapy. In clinical practice, elevated serum estrogen levels (luteinizing hormone) in females and high levels of 17β‐estradiol in males were significantly associated with improved survival outcomes in metastatic urothelial cancer patients receiving pembrolizumab.^[^ ^45a^ ^]^ Additionally, the antiestrogen ICI 182780 was found to upregulate integrin‐α5β1 expression and IL‐6 release, facilitating BCG internalization in BC cells and promoting the recruitment of monocytes and macrophages, thereby enhancing the efficacy of BCG therapy.^[^ ^45b^ ^]^ Estrogen can also attenuate the cytotoxicity of NK cells in various mouse models.^[^ ^181^ ^]^ In enzalutamide‐resistant PCa cells, NK‐mediated lysis was inhibited by the overexpression of ERα and Erβ.^[^ ^45c^ ^]^ However, this NK‐mediated lysis could be restored by fulvestrant, a selective estrogen receptor degrader, both in vitro and in vivo. Another randomized controlled trial assessed whether the estetrol produced by the fetal liver during pregnancy could mitigate the adverse effects of ADT in patients with advanced PCa.^[^ ^45d^ ^]^ The final results indicated that patients receiving both ADT and estetrol experienced significantly fewer hot flashes and reported higher health‐related quality of life scores compared to those undergoing ADT alone. This evidence suggests that estrogen and its receptors can significantly influence the treatment of BC and PCa. Estrogen not only enhances the efficacy of immunotherapy in breast cancer but also alleviates the side effects associated with ADT in patients with PCa. However, notably, many ER subtypes remain poorly understood in terms of their roles in tumor development and immune regulation. For example, ERα is androgen‐independent and may contribute to the emergence of CRPC.^[^ ^182^ ^]^ Therefore, the specific functions of ER subtypes must be elucidated to avoid the serious side effects induced by ER. **Figure** **4** shows the role of the ER in the development of PCa and BC. **Table** **2** shows the key references for ER in PCa and BC development.

**Table 2:** The key references of estrogen and estrogen receptor in prostate cancer and bladder cancer.

PMID	Organ	Stage	Cell line	Animal	Pathway/gene
23 204 329	Prostate	Development	PS‐1 and BPH‐1.	Tgln mice.	ERα
31 433 456	Prostate	Development	HuSLCs and DU145.	/	ERβ/MAPK and ERα/AKT.
27 918 915	Prostate	Development	/	C57BL/6 J mice and Shhtm2(cre/ERT2) Cjt/J mice.	β‐catenin and CCND1.
24 148 819	Bladder	Carcinogenesis	J82, 647v, and T24.	ERβKO mice.	ERβ/MCM5.
26 556 868	Bladder	Progression	647V, T24, and SV‐HUC.	Female nude mice.	ERβ/CCL2/CCR2/EMT/MMP9 pathway.
21 248 144	Prostate	Progression	WPMY‐1 and PC3.	/	ERα/TGF‐β1/MMP2 pathway
26 790 618	Prostate	Progression	RAW264.7, TRAMP‐C1, CWR22Rv‐1, C4‐2, and PC‐3.	B6 mice.	CCL5 and IL6.
26 045 993	Bladder	Progression	BCa T24, UMUC3, and 5637.	/	ERα/CCL1 and ERα/IL6 pathways.
29 684 419	Bladder	Progression	SV‐HUC, 5637 and 647 V.	Female nude mice.	ERβ/c‐MET or ERβ/IL‐1/c‐MET pathway.
31 455 760	Bladder	Progression	J82 and UMUC3.	Female mice.	ERα/circ_00 23642/miR‐490‐5p/EGFR pathway.
37 277 835	Bladder	Treatment	/	/	17β‐estradiol.
27 092 883	Bladder	Treatment	T24ERα and THP‐1.	FVB female mice.	ERα/IL6 and ERα/TNFα.
37 678 915	Prostate	Treatment	LNCAP and MDA‐PCa 2b.	NSG‐MHC I/II KO male mice.	ERs.

## 5 Conclusion and Perspectives

Sex disparities have always been an important topic in cancer development and management.^[^ ^183^ ^]^ Sex has been recognized to influence cancer immunity, thereby affecting the efficacy of immunotherapy in the current era of cancer treatment.^[^ ^184^ ^]^ Notably, the bladder and prostate share similar origins and functions, except for their reproductive roles. BC is classified as a “hot” cancer, whereas PCa is categorized as a “cold” cancer. In this context, we summarized and compared the immune differences between these two organs from a developmental perspective, utilizing sex hormones and their receptors as key indicators. Findings to date indicate that sex hormones and their receptors, AR and ER, significantly influence the composition and function of the immune microenvironment, thereby modulating prostate and bladder development, carcinogenesis, cancer progression, and treatment responses.

AR expression during embryonic development may reflect its role in PCa immune regulation in various cancers and cancer‐related cells. As mentioned previously, AR expression in different embryonic cells influences its function in various TME cells. Furthermore, AR‐related genes and pathways exhibited similar expression patterns during both the developmental stages and cancer progression in PCa and BC. These results suggest the need to explore the key genes and pathways involved in prostate and bladder development that may enhance immune regulation in PCa and BC. Investigating key genes or pathways during development can help mitigate the interference caused by the specificity and heterogeneity of tumors when studying them directly, thereby providing a valuable direction for cancer research. Consequently, the next step should involve a focus on the developmental differences between the bladder and prostate, identifying the genes or pathways that are the primary origins of immune differences. Moreover, both developing organs and tumors display rapid growth and active metabolism. However, development can regulate unlimited cell proliferation through programmed processes, leading to gradual differentiation. In contrast, tumors exhibit unlimited proliferation and dedifferentiation. Therefore, further research on the key genes or pathways that control growth and promote differentiation could validate the outcomes observed at different developmental stages of tumors, offering a crucial avenue for cancer immune research.

Sex hormones play a crucial role in sex‐related immune modulation. High‐dose testosterone has been shown to restore the cytotoxicity of peripheral blood polymorphonuclear neutrophils, which is inhibited by ADT‐derived TβRI in patients with PCa, thereby preventing PCa metastasis.^[^ ^185^ ^]^ Similarly, supraphysiological testosterone levels activate the cGAS/STING pathway by stimulating cytoplasmic nucleic acid sensors in LNCaP and LAPC4 cells, which recruit NK cells, CAMs, and neutrophils to the TME and induce immunogenic ferroptosis.^[^ ^186^ ^]^ Dihydrotestosterone also induces immune modulation and ferroptosis in LNCaP cells, but not in LAPC4 cells. The activation of the cGAS/STING pathway triggered by dihydrotestosterone is lower than that triggered by testosterone because of its rapid metabolism. However, another study indicated that high doses of dihydrotestosterone enhanced PD‐L1 expression in enzalutamide‐resistant C4‐2 cells via the AR/circFKBP5/miR‐513a‐5p axis, which inhibited NK cell cytotoxicit.^[^ ^13a^ ^]^ Furthermore, dihydrotestosterone promotes M1 polarization of CAMs by upregulating AR‐activated TNF‐related apoptosis‐inducing ligands in androgen‐resistant PCa cells, thereby enhancing the cytotoxicity of M1 CAMs both in vitro and in vivo.^[^ ^187^ ^]^ In PCa samples, treatment with estradiol or progesterone was associated with reduced M2 CAM infiltration compared to that in samples treated with dehydroepiandrosterone.^[^ ^3c^ ^]^ However, the specific functions of sex hormones in BC remain largely unknown. Current findings suggest that various types of sex hormones exhibit distinct immunomodulatory effects on specific tumor cells. This may be attributed to the ability of sex hormones to regulate not only nuclear AR but also other AR types located in different cellular compartments, such as the membrane AR receptors mentioned earlier, as well as other potential targets. The presence and functionality of these targets may vary depending on the tumor‐specific heterogeneity. Consequently, future research should not only emphasize the conditional roles of AR and ER subtypes within the immune microenvironment of BC and PCa but also consider the effects of sex hormones on additional receptors and pathways, as these factors may significantly influence therapeutic outcomes. Furthermore, endogenous sex hormones can be converted into other hormone types, which should be considered when modulating AR or ER function to enhance the effectiveness of AR‐ or ER‐based immunotherapies.

Previous studies have primarily focused on the roles of the AR and ER in cancer cells. However, the advent of single‐cell sequencing has enabled us to explore the exploration of regulatory networks within various TME cells.^[^ ^188^ ^]^ The diverse functions of ERα in both cancer and stromal cells serve as a prominent example, inspiring further investigation into the specific roles of ER subtypes across different cell types.^[^ ^171^, ^175^ ^]^ This highlights the need to directly regulate immune cell composition and function to manage PCa and BC effectively. These findings pave the way for enhancing the efficacy of current therapies that utilize AR/ER medications, such as immunotherapy and ADT.^[^ ^45b^ ^]^ Furthermore, the interactions between the AR and ER significantly affect the immune microenvironment by regulating the MMP family.^[^ ^148^, ^166^ ^]^ In ADT, ERα contributes to the emergence of CRPC due to androgen independence.^[^ ^182^ ^]^ However, there is a notable lack of research on the interactions between AR and ER in immunity against BC and PCa. However, the dynamics of this interaction during development remain unclear and require further investigation.

Phytoestrogen administration attenuates the incidence of PCa.^[^ ^189^ ^]^ Furthermore, phytoestrogen administration influence the prognosis of patients with PCa or BC.^[^ ^190^ ^]^ A randomized controlled trial (NCT02759380) demonstrated that the consumption of phytoestrogens by PCa patients affects serum estradiol concentrations in the ERβ subgroup.^[^ ^191^ ^]^ Phytomedicine has been shown to manage urological cancers by regulating the immune microenvironment.^[^ ^192^ ^]^ For instance, *Yerba mate*, a type of infusion, has anti‐inflammatory effects.^[^ ^193^ ^]^ *Yerba mate* extract attenuates the proliferation of PC cells by suppressing Erα.^[^ ^194^ ^]^ Therefore, when managing patients with PCa and BC, as well as in daily life, it is essential to carefully consider the role of estrogen in the human body, particularly when selecting food or medication, as these factors contribute to the development of personalized nutritional oncology. This necessitates a clear understanding of the specific roles of estrogen and its receptor subtypes in tumor immunity. By comprehending these mechanisms, we can better regulate food and medication intake or coadminister estrogen‐modulating drugs along with existing treatments.

Membrane sex hormone receptors, including ER and AR, play a significant role in the development and progression of PCa and BC.^[^ ^64^, ^159^ ^]^ For example, in the prostate, YAP overexpression has been documented in benign prostatic hyperplasia and is associated with lower levels of estrogen and GPER expression.^[^ ^160^ ^]^ Membrane sex hormone receptors primarily mediate rapid, nongenomic responses by activating intracellular signaling cascades, such as MAPK and PI3K/Akt. In contrast, nuclear sex hormone receptors influence long‐term genomic effects through direct interactions with DNA, thereby affecting gene transcription and protein synthesis.^[^ ^195^ ^]^ Membrane receptors are crucial for acute cellular responses, whereas nuclear receptors regulate sustained physiological processes, including reproductive functions and secondary sexual characteristics.^[^ ^196^ ^]^ Future research should not only explore the roles of each hormone receptor subtype within the cell nucleus across various cell types but also examine but also compare the types and specific functions of membrane hormone receptors. Analyzing the molecular interaction mechanisms will lay the groundwork for investigating and leveraging nuclear and membrane hormone receptors in the immune regulation of PCa and BC.

In conclusion, from a developmental perspective, by aggregating and analyzing existing data, we demonstrated that PCa and BC activate genes and pathways similar to those observed during development. However, significant gaps remain in the literature, particularly concerning the study of sex hormones and their receptors in the bladder. The next step must involve investigating the key genes and pathways associated with bladder development to identify primary immune regulatory similarities and differences. Fortunately, numerous innovative and powerful techniques have been developed, including CRISPR,^[^ ^197^ ^]^ single‐cell sequencing,^[^ ^198^ ^]^ biomaterials,^[^ ^199^ ^]^ biomembrane probes,^[^ ^200^ ^]^ and artificial intelligence.^[^ ^201^ ^]^ In addition, the recently awarded Nobel Prize in Chemistry for advancements in protein structure may enhance our understanding of specific protein interaction mechanisms. Techniques such as single‐cell spatial transcriptomics, metabolomics, and proteomics, enabled by single‐cell technology, can facilitate the rapid and precise exploration of the roles of sex hormones and their receptors within individual cells and their effects on the immune microenvironment.^[^ ^202^ ^]^

## Conflict of Interest

The authors declare no conflict of interest.

## Author Contributions

D.X.L., Z.P.W., Q.X.Y., J.W., and R.C.W. contributed equally to this work. D.X.L., Z.P.W., Q.X.Y., J.W., and R.C.W. proposed the project, conducted data analysis, interpreted the data, and wrote the manuscript. D.X.L., Z.P.W., Q.X.Y., J.W., and R.C.W. conducted data analysis, interpreted the data. W.C.C., W.R.W., and D.C.F. supervised the project, and interpreted the data. All authors reviewed and edited the manuscript.
